# Supplementary material for: Identification and functional analysis of non-coding regulatory small RNA FenSr3 in Bacillus amyloliquefaciens LPB-18
Source: PeerJ. 2023 May 15;11:e15236. doi: 10.7717/peerj.15236 (PMC10194069; doi:10.7717/peerj.15236)
Supplement: Supplemental Information 4 [file peerj-11-15236-s004.zip › KO/CK-vs-T1_map/map04672.html]

KEGG PATHWAY: Intestinal immune network for IgA production - Reference pathway


|  |  |
| --- | --- |
| **Intestinal immune network for IgA production - Reference pathway** |  |

[
Pathway menu
| Organism menu
| Pathway entry
| Show description
| User data mapping
]

The intestine is the largest lymphoid tissue in the body. One striking feature of intestinal immunity is its ability to generate great amounts of noninflammatory immunoglobulin A (IgA) antibodies that serve as the first line of defense against microorganisms. The basic map of IgA production includes induction of mucosal B cells in the Peyer's patches, circulation through the bloodstream and homing to intestinal mucosa of IgA-commited plasma cells, and local antibody production for export across the intestinal membranes. Multiple cytokines, including TGF-{beta}, IL-10, IL-4, IL-5, and IL-6, are required to promote IgA class switching and terminal differentiation process of the B cells. Secreted IgA promotes immune exclusion by entrapping dietary antigens and microorganisms in the mucus and functions for neutralization of toxins and pathogenic microbes.

|  |  |  |
| --- | --- | --- |
| Reference pathway | 184% 150% 122% 100% 82% 67% 55% | 图片下载 |
